# Attention-assisted hybrid 1D CNN-BiLSTM model for predicting electric field induced by transcranial magnetic stimulation coil

**DOI:** 10.1038/s41598-023-29695-6

**Published:** 2023-02-13

**Authors:** Khaleda Akhter Sathi, Md Kamal Hosain, Md. Azad Hossain, Abbas Z. Kouzani

**Affiliations:** 1 Department of Electronics and Telecommunication Engineering, Chittagong University of Engineering & Technology, Chittagong, 4349 Bangladesh; 2 Department of Electronics & Telecommunication Engineering, Rajshahi University of Engineering & Technology, Rajshahi, 6204 Bangladesh; 3 School of Engineering, Deakin University, Geelong, VIC 3216 Australia

**Keywords:** Computational models, Computational neuroscience, Machine learning

## Abstract

Deep learning-based models such as deep neural network (DNN) and convolutional neural network (CNN) have recently been established as state-of-the-art for enumerating electric fields from transcranial magnetic stimulation coil. One of the main challenges related to this electric field enumeration is the prediction time and accuracy. Despite the low computational cost, the performance of the existing prediction models for electric field enumeration is quite inefficient. This study proposes a 1D CNN-based bi-directional long short-term memory (BiLSTM) model with an attention mechanism to predict electric field induced by a transcranial magnetic stimulation coil. The model employs three consecutive 1D CNN layers followed by the BiLSTM layer for extracting deep features. After that, the weights of the deep features are redistributed and integrated by the attention mechanism and a fully connected layer is utilized for the prediction. For the prediction purpose, six input features including coil turns of single wing, coil thickness, coil diameter, distance between two wings, distance between head and coil position, and angle between two wings of coil are mapped with the output of the electric field. The performance evaluation is conducted based on four verification metrics (e.g. R2, MSE, MAE, and RMSE) between the simulated data and predicted data. The results indicate that the proposed model outperforms existing DNN and CNN models in predicting the induced electrical field with R2 = 0.9992, MSE = 0.0005, MAE = 0.0188, and RMSE = 0.0228 in the testing stage.

## Introduction

The neurological disorders including major depression, traumatic brain injury, parkinson’s disease, and post-traumatic stress are commonly treated with transcranial magnetic stimulation (TMS) therapeutic technique as it provides an effective outcome than other therapeutic techniques^1–4^. The magnetic stimulation technique (i.e., TMS) uses a magnetic coil that is placed on the patient’s head. The magnetic coil is activated by applying a high-valued external current pulse with a short period of time^5^. The external applied current pulse produces a magnetic field at the surface of the coil that results in an electric field in the conductive brain tissues of the human head^6^. The induced electric field then forms an axial depolarization in the abnormal neural tissues that are responsible for neural disorders^7^. To activate the targeted abnormal neurons with optimum therapeutic effectiveness, the induced electric field needs to be strong enough at the target side than the other non-targeted region^8,9^. In addition to the strength of the electric field, the depth and focality of the induced electric field are the main concerns in effective TMS treatment.

In TMS therapy, both single coil and assembly coils are used. The single TMS coil has drawbacks associated with the focality and depth of the induced electric field, as it cannot provide an optimum tradeoff of these two parameters. The commercially available single TMS coils including figure of eight, halo, V, circular, H, and double cone coils have been suffering the focality-depth tradeoff problem. All of these single coils except H coil use circular-shaped geometry to produce a focal electric field at the targeted side. Whereas, the H coil generates an electric field with a lower focality stimulation at the target side. For this reason, recent researches and clinical trials are focused on the assembly-type deep TMS (dTMS) coils to maintain focality-depth tradeoff at the target side^10–13^. Among these dTMS coils, the H7 coil^13^ dominates the others in terms of focality-depth tradeoff performance. Despite having the superior focality depth tradeoff performance, there is a difficulty related to coil geometry which results in the likelihood of changing trade-offs with varying coil positions. Moreover, the expense of higher and spreading induced electric field at the superficial cortical region can cause serious headaches. By considering this, a halo-V assembly (HVA) coil is developed^14^ to provide optimum therapeutic efficacy by ensuring the finest tradeoff at the target side and reducing the expense of stimulation at the non-targeted superficial cortical region. Furthermore, assembly coil has more design parameters than single coil to achieve desired focality-depth tradeoff. Thereby, for conducting new research and finding a new clinical application of the TMS, the electric field of the assembly coils needs to be enumerated and analyzed with the commercially available electromagnetic (EM) simulation software such as SimNIBS, Sim4life, and COMSOL Multiphysics. This task requires a human head model as a conductive medium and a coil placed on the human head model to determine the induced electric field. The required time to design the new assembly coil and human head model and the simulation time to enumerate induced electric field is huge (e.g. computational cost) that tends to be hours or a day-long^15–19^. Considering this fact, deep learning (DL) based models that are normally dependent on the data are used to enumerate EM field values by reducing the computational cost^20^. Commonly used tasks including segmentation, classification, regression, and clustering are performed in the DL model^21–32^. The electric field estimation is normally based on the regression task that utilizes a deep neural network (DNN) based DL model. In the DL model, a complex non-linear relationship between input and output is developed by adding multiple hidden layers^33^.

Recently, some researches are focused on DL-based electric field enumeration techniques from TMS coils in human tissue models. For instance, Yokota et al.^15^ proposed a DL model called as U-Net to determine the electric field from a figure of eight TMS coil by considering one coil parameter such as coil position. The database comprised both input and output parameters as the U-Net regressed the electric field in a supervised learning manner. The output parameter such as electric field was simulated in SimNIBS software where the volume head model was imported from FreeSurfer segmentation software. The FreeSurfer software segmented each part of the brain regions from a brain magnetic resonance image (MRI) to generate a volume head model. After creating the database, it was feeded to the 3D U-Net model for training, and finally, the electric field was predicted from test data based on the model training performance. The encoder and decoder-based U-Net model performed electric field prediction with an accuracy of 93%. In another study, Afuwape et al.^16^ utilized a convolutional neural network (CNN) based DL model to predict electric fields from sixteen different single-type TMS coils. Initially, the creation of the dataset was performed in Sim4Life software by simulating the different coils on the 3D human head that was generated from T1-weighted MRI. After that, the training of the created dataset was performed on a 3D deep CNN model that regresses the output electric field from the feed input dataset. The performance of the model in predicting electric field was found 92% accurate. Moreover, Sathi et al.^17^ proposed an electric field enumeration model based on DNN. The model train and test dataset were created from COMSOL Multiphysics 5.0a software that generated input data from a two-shell human head model and coil geometric structure. In addition, the target data were generated after simulating the model for six coil designing parameters of the HVA coil. The training and testing of prediction model were conducted on 100 datasets. The model electric field prediction accuracy on the testing dataset was found approximately 76%.

In terms of computational time, all existing DL-based models have the capability of computing electric fields in a lower period of time. However, the data creation processes from the segmentation software presented in Refs.^15,16^ are quite challenging because it requires high contrast T1-weighted MRI brain image. It is also difficult to produce an actual human head model from low-contrast MRI images. Moreover, the time requirement for the segmentation process is also a problem. Another delimitation of these models is that the electric field estimation accuracy depends on the quality of the MRI scans that result in performance deterioration if the picture quality deteriorates^34^. The utilization of a deep network for both encoding and decoding of data can increase time during training. Furthermore, the use of a single coil parameter such as coil position as the input parameter cannot aid a value to effectively determine the induced electric field. In addition, the prediction accuracy of the electric field of the model developed in Ref.^17^ is quite low which may cause incorrect estimation of the new unknown data.

By considering all limitations such as the dependency of image quality in 3D DL network^15,16^ and low prediction accuracy in DNN model^17^, a new attention-assisted hybrid 1D CNN-BiLSTM model is proposed in this work for predicting electric field induced by TMS coil. The novelty of this work is to develop a new 1D DL model that use numeric data in electric field enumeration as well as to improve enumeration accuracy than the existing 3D and 1D based state-of-the-art models. In this work, an assembly coil with several designing parameters rather than single parameter i.e., coil position is considered. The model regresses the output electric field from continuous numerical data including different parameters of HVA coil to learn the model from these sample data for final prediction. The created 100 samples dataset is fed to the deep DL model to provide complexity and variation of the dataset and find out the relativity of the input data that are responsible for producing output data of electric field by calculating the attention between each of the input features. Finally, the trained regression model predicts a continuous electric field value from testing data that are unknown to the trained model. The main advantage of the model is associated with the improved prediction accuracy than the recently reported DL-based electric field enumeration model and reduced computational time.

The primary contributions of this work are outlined as follows:

- A hybrid CNN and BiLSTM based framework is proposed for the prediction of induced electric field in HVA TMS coil under low-frequency and high amplitude current pulse conditions.
- An attention mechanism for high accuracy is adopted by redistributing the weights of the deep features extracted by CNN-BiLSTM model.
- The prediction results of CNN-BiLSTM-Attention model with state-of-the-art models are compared. The proposed model enhances performance in both prediction accuracy and computational time complexity.

The remaining part of the paper is organized as follows: In “Methods” section, the dataset creation and architecture of the CNN-BiLSTM-Attention model have been expressed in detail. The results and analysis of model performance in terms of prediction accuracy are described in “Results and performance evaluation” section. Finally, in “Conclusion” section, the concluding remarks is included with future directions briefly.

## Methods

**Figure 1:**
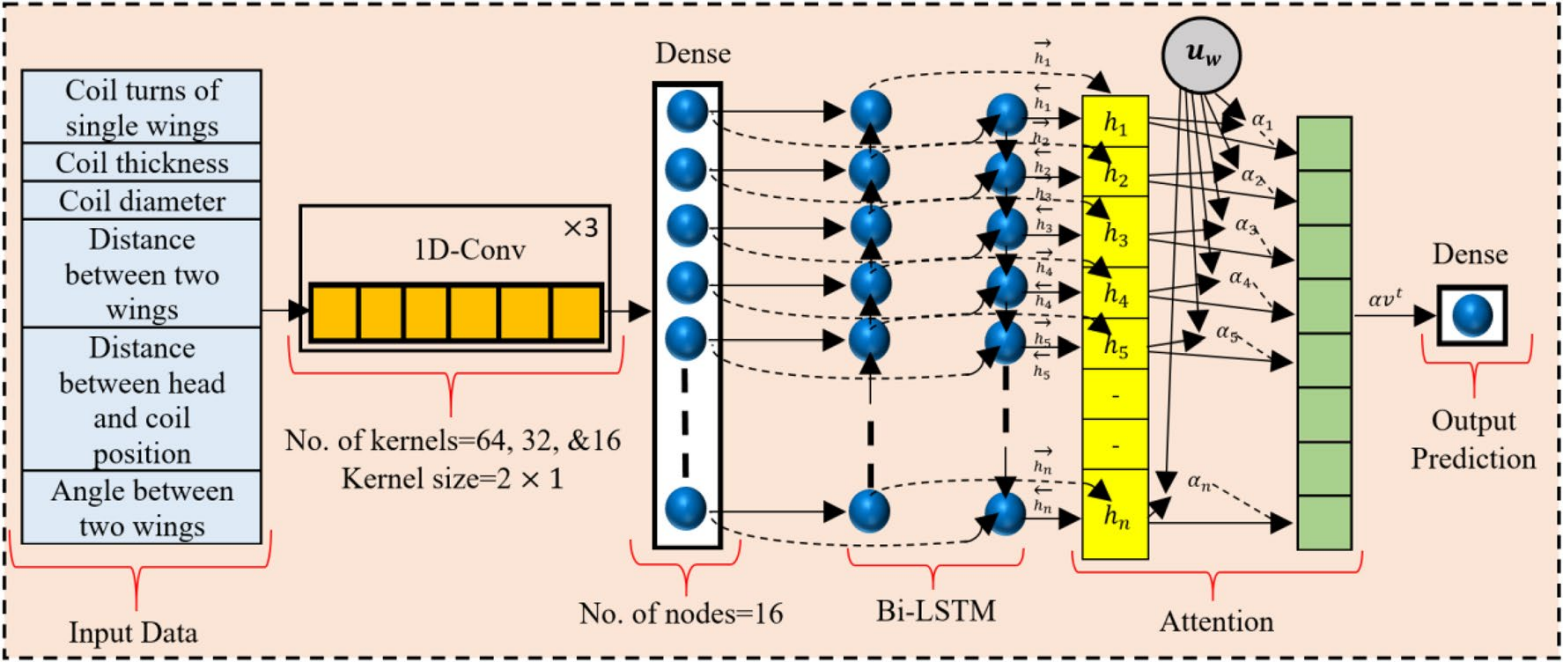
Overview of the proposed electric field prediction network architecture comprising attention mechanism in the hybrid 1D CNN-BiLSTM model.

The architecture of the proposed attention-based hybrid 1D CNN-BiLSTM model for electric field prediction is shown in Fig. 1. The process of prediction is started by generating a dataset with the composition of input and target features. Here, the input features are the composition of six coil design parameters of the HVA coil and the target feature is the induced electric field. After that, the raw dataset is fed into 1D CNN network for feature extraction by applying three consecutive 1D Conv layers with a kernel size of 1 $\times$ 2. Subsequently, a fully connected layer with 16 nodes is used for the vectorial representation of the features. Then, the sequential model called Bi-LSTM is utilized to learn the valid information from the feature maps by using forward and backward hidden states. Later on, an attention layer is performed that merged important features and chooses the critical features by redistributing the weights. Finally, a fully connected layer is used for electric field prediction. Moreover, the overall processes of the electric field prediction are described below in detail.

### Configuration of HVA coil with human head model

**Figure 2:**
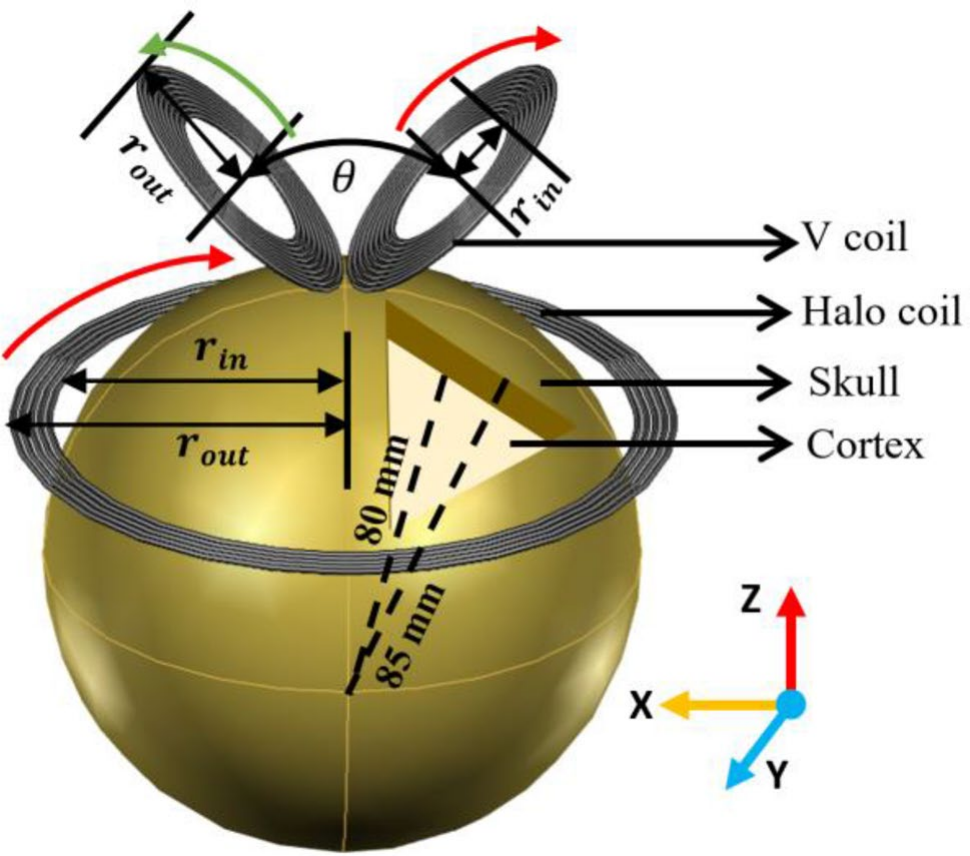
An illustration of HVA coil configuration with two shell human head model. The clockwise and anti-clockwise directions of coil current are indicated as red and green arrow line respectively.

A 3D visualization of a HVA coil with two-shell human head model in the cartesian coordinate system is illustrated in Fig. 2. The human head model consists of two layers of tissues including skull and cortex. The radii of the outer and inner layers are 85 mm and 80 mm respectively. On the contrary, the HVA coil is comprised of two single coils such as V-coil and halo coil. The assembly coil has a total of 23 turns among them 5 for the halo coil and 9 for each wings of the V coil. In the HVA coil, the V coil is placed 5 mm above the vertex of the head model and the halo coil is placed 90 mm below the vertex of the head model. All the design parameters of HVA coil are summarized in Table 1. After designing the coil configuration with a torus shape, the copper material with an electrical conductivity of 5.8 × 10^7^ S/m is set. In addition, the electromagnetic properties of materials selected for the human head model and coil are listed in Table 2^35,36^.

**Table 1:** Design parameters of HVA coil.

Coil name		Inner radius ${r}_{in}$ (mm)	Outer radius ${r}_{out}$ (mm)	Total coil turns	Angle between two wings $\theta$ (°)
HVA	V	27.5	47.5	18	45°
	Halo	87.5	97.5	5	–

**Table 2:** Electromagnetic properties of HVA coil and two-shell human head model at an operating frequency of 2500 Hz.

Material	Conductivity [S/m]	Relative permittivity	Relative permeability
Skull	0.02	30,380	1
Cortex	4	80	0.99
Copper	5.8 $\times \,{10}^{7}$	1	0.99

The electric field in the brain tissues is generated with the HVA coil by applying a current pulse with a high amplitude of 5000A^16^ and low-frequency of 2500 Hz. Based on the theory of electromagnetic induction, the varying magnetic field is produced by the rate of change of current in the HVA coil. The electric field^37^ is induced in the brain conductive medium by the time-varying magnetic field according to Faraday’s law of induction (1). Equation (1) can be expressed in terms of magnetic vector potential $\mathbf{A}$, instead of magnetic field $\mathbf{B}$ as shown in (2). The vector potential $\mathbf{A}$ can be expressed as (3): where **r** is the distance from the HVA coil to the field point and $d{\varvec{l}}$ is the vector tangent to the coil at the point.

1 $$\oint \limits_{c}\mathbf{E}\cdot d{\varvec{l}}=-\int \limits_{s}\frac{\partial \mathbf{B}}{\partial t}\cdot d{\varvec{s}},$$

2 $$\mathbf{B}=\nabla \times \mathbf{A},$$

3 $$\mathbf{A}=\frac{{\mu }_{0}I}{4\pi }\int \frac{1}{{\varvec{r}}}\cdot d{\varvec{l}}.$$

In the neurons, the induced electric field, $\mathbf{E}$ is the sum of primary electric field component ${\mathbf{E}}_{1}$ and secondary component ${\mathbf{E}}_{2}$. The ${\mathbf{E}}_{1}$ is responsible for the time-varying magnetic field from HVA coil and ${\mathbf{E}}_{2}$ results from the accumulation of charges on the brain interface as expressd in (4).

4 $$\mathbf{E}={\mathbf{E}}_{1}+{\mathbf{E}}_{2}=-\frac{\partial \mathbf{A}}{\partial t}-\nabla \mathbf{V}.$$

Using Eq. (3), $\mathbf{E}$ can be expressed as (5). Where, the minus sign specifies that the direction of the induced electric field is opposite to the direction of the applied current in the HVA stimulating coil. Therefore, the induced electric field is the rate of change of current $\left(\frac{dI}{dt}\right)$ in the HVA stimulating coil.

5 $$\mathbf{E}=-\frac{{\mu }_{0}}{4\pi }\frac{dI}{dt}\int \frac{1}{{\varvec{r}}}\cdot d{\varvec{l}}.$$

Based on the physics described here, the value of $\mathbf{E}$ is evaluated on COMSOL Multiphysics 5.0a software. During the simulation, the overall 3D model including the two-shell human head and HVA coil are divided into several sub-domains with free tetrahedral meshing elements for solving the governing equations which permit accurate numerical results in a low computational time.

### Dataset creation

**Figure 3:**
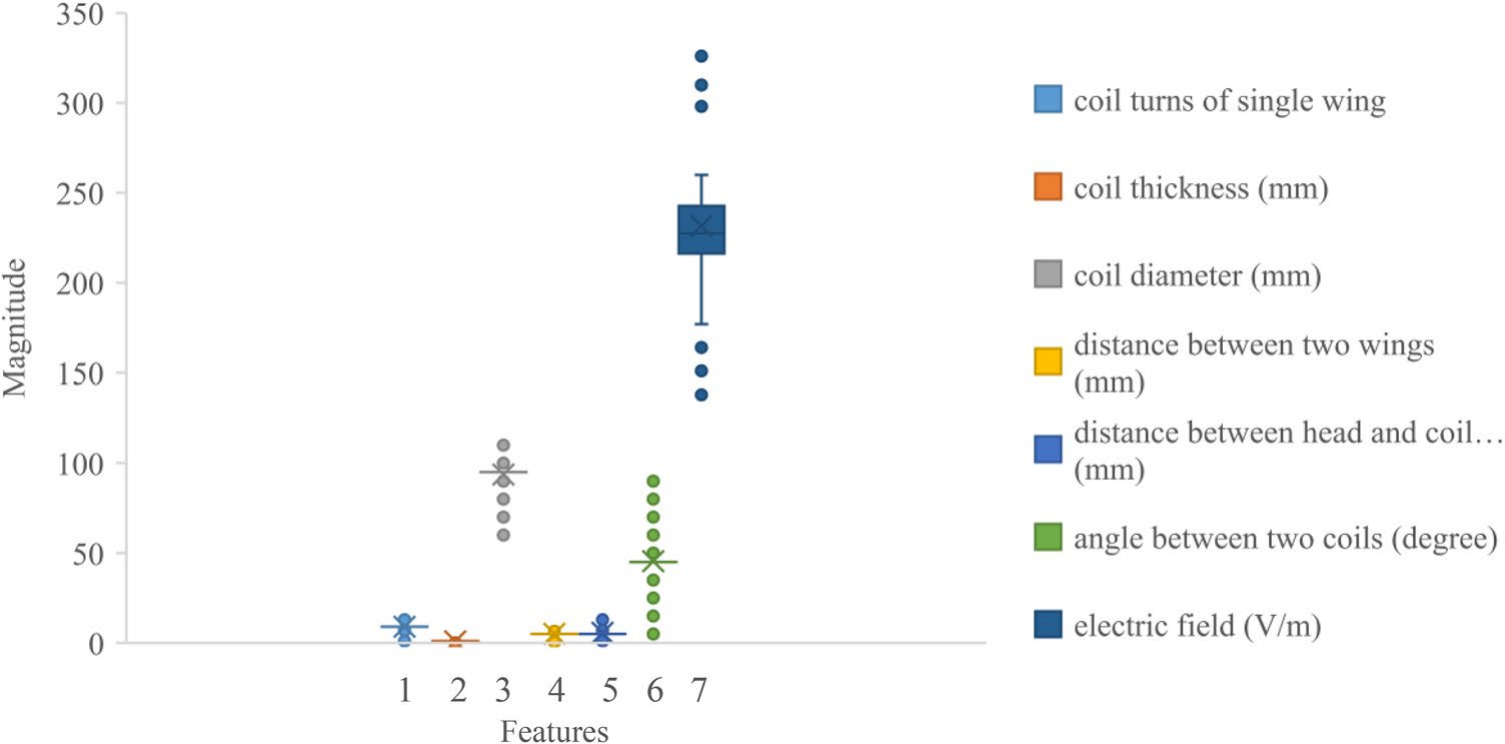
The boxplot of original feature visualization.

A total of N = 100 data samples are collected from COMSOL Multiphysics software and formatted into a .CSV file for processing with the proposed attention-based 1D CNN-BiLSTM model. The N samples create the dataset, represented as D: = ${\left\{h, {[{x}_{1};{x}_{2};\dots .;{x}_{6}]}_{n},{E}_{n}\right\}}_{n=1}^{N}$ with the combination of input ${\left\{h, {[{x}_{1};{x}_{2};\dots .;{x}_{6}]}_{n}\right\}}_{n=1}^{N}$ and output ${\left\{{E}_{n}\right\}}_{n=1}^{N}$ data under the low frequency of 2500 Hz and high amplitude of 5000 A current pulse condition. Where, the output induced electric fields, ${E}_{n}$ are found from six different HVA coil designing parameters including coil turns of single wing (${x}_{1}$), coil thickness (${x}_{2}$), coil diameter (${x}_{3}$), distance between two wings (${x}_{4}$), distance between head and coil position (${x}_{5}$), and angle between two wings (${x}_{6}$) on the human head model. The values of each input parameter and corresponding output variations are summarized in Table 3. Moreover, the box-plot distributions of all input and output parameters are presented in Fig. 3. In this figure, the median values of two input parameters such as coil diameter and angle between two coils are above 50 which designate the dominating factors in calculating the electric field values.

**Table 3:** Representation of input and output features.

Inputs and outputs	Features to form model (unit)	Range
Input	Coil turns of single wing	1–12
	Coil thickness (mm)	0.1–0.9
	Coil diameter (mm)	60–110
	Distance between two wings (mm)	0.5–10
	Distance between head and coil position (mm)	1–15
	Angle between two wings (°)	5–90
Output	Electric field (V/m)	130–300

### Proposed attention based 1D CNN-BiLSTM prediction model

After creating the dataset, the preprocessing technique such as normalization is employed before feeding into the final prediction model. The normalization technique smooths the training time of the network by converting all numerical values in the range of 0 and 1. Then, the normalized data are used for further processing through the proposed prediction model that follows the following sequential steps.

#### 1D CNN model

The utilization of 1D CNN model on the normalized continuous numerical data can extract a representative and effective feature by performing a one-dimensional convolution operation with multiple filters. To match the one-dimensional characteristics of the continuous numerical data, the 1D convolutional filters and feature maps are employed with the CNN model. By applying more than one convolutional layers, the 1D CNN model deepens the feature extraction process. Thus a higher level of features makes the prediction task more robust and discriminative. Initially, the preprocessed normalized data with a shape of (6 × 1) is used as the input of the 1D CNN model. Then, the input data is passed through three consecutive 1D convolutional (Conv) layers to extract deep features, where each of the Conv layers contains 64, 32, and 16 1D kernels respectively with a size of (2 × 1). After performing the convolution operation, a rectified linear unit (ReLU) activation function is utilized which introduces non-linearity in the model and reduces the overfitting problem as well. The operation of the 1D Conv layer followed by ReLU activation function is performed as follows:

6 $${{y}_{j}^{(k)}=ReLU}_{\mathrm{1,2},3\in h}\left[\sum \limits_{i=1}^{{N}_{(k-1)}}Conv1D\left({x}_{i}^{(k-1)},{W}_{i,j}^{(k)}\right)+{b}_{j}^{(k)}\right],$$

where, the *i*th feature map in the (*k* − 1)th layer is represented as ${x}_{i}^{(k-1)}$ and the *j*th feature map in the *k*th layer is represented by ${y}_{j}^{k}$. Moreover, the trainable convolutional kernel is denoted as ${W}_{i,j}^{(k)}$ and ${N}_{(k-1)}$ denotes the total number of feature maps in the (*k* − 1)th layer. The 1D convolution operation without zero-padding is performed on ${x}_{i}^{(k-1)}$ and ${W}_{i,j}^{(k)}$. Therefore, the dimension reduction of the feature maps is found at the (*k* − 1)th layer than the *k*th layer. In addition, the bias of the *j*th feature map in the *k*th layer is represented as ${b}_{j}^{(k)}$. The operation of ReLU activation function is performed as follows:

7 $$ReLU\left({P}_{h}\right)={\text{max}} \left(0, {P}_{h}\right),$$

where,

8 $${P}_{h}=\sum \limits_{i=1}^{{N}_{(k-1)}}Conv1D\left({x}_{i}^{(k-1)},{W}_{i,j}^{(k)}\right)+{b}_{j}^{(k)}.$$

After passing through all 1D convolutional layers, the obtained 16 feature maps with the size of (4 × 1) are fed into one dense layer with 16 nodes. Then, the output features are fed into the Bi-LSTM model for finding valid information from the feature maps.

#### Bi-directional LSTM model

As the dataset is composed of some sort of noisy numerical data, the prediction of the electric field from the noisy data is complex. For estimating electric field and eliminating the noise, the Bi-LSTM network is adopted to the 1D CNN model. The Bi-directional LSTM network is a two-way stacked LSTM network with forward and backward LSTM features. The previous values are learned through the forward LSTM network, and similarly, the future values are learned in the reverse direction by applying a backward LSTM network. The utilization of hidden states in the latter layer help to learn both forward and backward information. The mathematical equation for performing this task through the Bi-LSTM unit is explained as follows:

9 $$\overrightarrow{{h}_{n}}=\sigma \left({W}_{1}{x}_{t}+{W}_{2}\overrightarrow{{h}_{n-1}}\right)\times {\text{tan}}h\left({C}_{t}\right),$$

10 $$\overrightarrow{{h}_{n}}=\sigma \left({W}_{3}{x}_{t}+{W}_{4}\overleftarrow{{h}_{n-1}}\right)\times \mathrm{tanh}\left({C}_{t}^{^{\prime}}\right),$$

11 $${h}_{n}^{[p]}={{W}_{5}\overrightarrow{{h}_{n}}+W}_{6}\overleftarrow{{h}_{n}},$$

where, ${x}_{t}$ is the input at time *t*. *W*’s are the weights of gates of LSTM cells. $\overrightarrow{{h}_{n}}$ and $\overleftarrow{{h}_{n}}$ are the forward and backward outputs, respectively. The generated Bi-LSTM features from the *i*th features in the *p*th layer is denoted as ${h}_{n}^{[p]}$ that keeps information in Bi-directional steps. In this paper, the generated feature vectors of 1D CNN and dense are fed to the Bi-LSTM model. An activation function of ‘tan*h*’ is adopted to finish the normalization and help to reduce the overfitting problem.

#### Attention mechanism

Generally, all the parameters of the input data do not contribute equally to decide whether the parameter belongs to a particular prediction. Therefore, the utilization of the attention mechanism is performed to emphasize the most important parameters during prediction. In the attention mechanism, the weight is ${\alpha }_{n}$ for each individual Bi-LSTM feature, and ${h}_{n}^{[p]}$ is assigned with a focus on output labels. Mathematically, the followings are computed for the attention function:

12 $${\mu }_{i}={\text{tan}}h\left({W}_{d}\times flatten \left({h}_{n}^{\left[p\right]}\right)+b\right),$$

13 $${\alpha }_{n}= \frac{{\text{e}}{\text{xp}}({\mu }_{i} {u}_{w})}{\sum_{n}{\text{exp}}({\mu }_{i} {u}_{w})},$$

14 $$\alpha {v}^{t}=\sum_{n}{\alpha }_{n} {h}_{n}^{\left[p\right]}.$$

Here, ${h}_{n}^{\left[p\right]}$ is the feature vector obtained in the BiLSTM layer, which is passed to a one-layer neural network (*p* = 1) to get the ${\mu }_{i}$ as a hidden representation of ${h}_{n}^{\left[p\right]}$. The ${W}_{d}$ and *b* are weight matrix and bias vector respectively that are initialized during the neural network training. The influences of the important parameters can be measured by calculating the similarity between ${\mu }_{i}$ and ${u}_{w}$. Afterward, a normalized weight, ${\alpha }_{n}$ is obtained by using the softmax function for each input feature. Finally, the attentive feature $\alpha {v}^{t}$ is fed to a dense layer consisting of one neuron. The predicted output, ${E}_{Pred}$ can be represented as:

15 $${E}_{Pred}=Linear \left(\sum_{j}{W}_{kj}\times \alpha {v}_{j}^{t}+ {b}_{k}\right),$$

where, ${W}_{kj}$ and ${b}_{k}$ are represented as the weight matrix, and bias vector respectively. The activation function ‘*linear*’ is added to the proposed model for final electric field prediction. The properties of entire network with their specification and the number of required parameters are demonstrated in Table 4. In this work, the attention-based model with 6641 trainable parameters acquire the best performance for the prediction of the induced electric field.

**Table 4:** Layer properties of proposed attention-based hybrid 1D CNN-BiLSTM model.

Layer no.	Layer name	Feature size	Specification	Parameter
1	Input	6 $\times$ 1		0
2	1D Convolutional	6 $\times$ 64	Filter size: 2 $\times$ 1 Filter number: 64	192
3	1D Convolutional	5 $\times 32$	Filter size: 2 $\times$ 1 Filter number: 32	4128
4	1D Convolutional	4 $\times$ 16	Filter size: 2 $\times$ 1 Filter number: 16	1040
5	Dense	4 $\times$ 16	–	272
6	Bi-LSTM	4 $\times$ 8	Hidden units: 4	672
7	Attention	16	Units: 16	320
8	Dense	1	–	17
				Total = 6641

### Prediction model validation

To validate the proposed attention-based hybrid 1D CNN-BiLSTM model, the tenfold cross-validation method is utilized that randomly divides the overall data into ten approximately equal sections. Then, each time, one of the splitted sections is selected as the test set, and the rest of the sections are considered as the training set. At every iteration, the model is trained using data shuffling. Finally, the estimation of the model evaluation matrices is performed by taking an average of ten predicted results. The summary of the ten-fold cross-validation methods is depicted as follows:

- Partitioning the raw dataset into ten parts containing an equal number of numerical values in each part.
- One part is selected as the test set for each iteration, and the remaining dataset is utilized as a training set for the model.
- After training, the results of ten iterations are averaged to obtain the final test results.

The selection of optimum hyperparameter values is important to train the proposed prediction model for the superior results. Table 5 represents the optimal values of the hyperparameters of the proposed attention-assisted hybrid 1D CNN-BiLSTM model. The model is compiled using an Adam optimizer with an initial learning rate of 0.01. Moreover, the model training is performed for 100 epochs per fold. The mean absolute error is chosen as a loss function to compute model loss. For conducting training and testing of the proposed model, the Google Colab platform is used with Python version 3.7.13. The model is implemented using Keras = 2.8.0 and TensorFlow = 2.8.2 framework. The Pandas = 1.3.5 and Sklearn = 1.0.2 packages have been used for data preparation and evaluation respectively. During the training, the model occupied 1.43 GB of RAM and 38.58 GB of disk space in the Colab environment.

**Table 5:** Hyper-parameters setting of the proposed model.

Hyper-parameter	Value
Loss function	Mean absolute error
Initial learning rate	0.01
Epochs	100
Optimizer	Adam

## Results and performance evaluation

The quantitative analysis of the attention-assisted 1D CNN-BiLSTM prediction model is performed based on four verification matrices including coefficient of determination (R^2^), mean squared error (MSE), mean absolute error (MAE), and root mean squared error (RMSE). The ${R}^{2}$ metric evaluates the quality of regression model by measuring how well the model can predict electric field data from the unknown input data. The preferable value of ${R}^{2}$ in prediction task is considered to be approximately equal to 1. On the other hand, the evaluation metric, MSE is also known as the regression loss function that measures the loss by summing the square of the differences between the predicted value and actual value. Since the loss needs to be lowest, the standard value of MSE for the regression task should be equal to 0. Another loss function called, MAE is measured by averaging all the absolute error values. Similar to the MSE, the model with a MAE value of approximately 0 is preferable. Moreover, the value of RMSE of 0 is desirable for prediction model as it calculates loss by the square root of the sum of square deviation of actual and predicted values over the total number of data, *n*. When the approximate value of the RMSE is more close to 0, it means more perfectly fitted the predicted values to the actual values. The mathematical representations of four above-mentioned metrics are expressed in Eqs. (16) to (19). Where, the true output and predicted output are indicated by ${y}_{true}$ and ${y}_{pred}$ respectively. In addition, the mean value of the ground truth output is represented as ${y}_{mean}$ and the total number of data is represented as n.

16 $${R}^{2}=1-\frac{\sum_{k=1}^{n}{\left({y}_{true}-{y}_{pred}\right)}^{2}}{\sum_{k=1}^{n}{\left({y}_{true}-{y}_{mean}\right)}^{2}}\in \left[0, 1\right],$$

where $${y}_{mean}=\frac{1}{n}\sum_{k=1}^{n}{y}_{true}.$$

17 $$MSE = \frac{\sum_{k=1}^{n}{({y}_{true}-{y}_{pred})}^{2}}{n},$$

18 $$MAE =\frac{\sum_{k=1}^{n}\left|{y}_{true}-{y}_{pred}\right|}{n} \in \left[0, +\infty \right],$$

19 $$RMSE =\sqrt{\frac{\sum_{k=1}^{n}{({y}_{true}-{y}_{pred})}^{2}}{n}}\in \left[0, +\infty \right].$$

**Figure 4:**
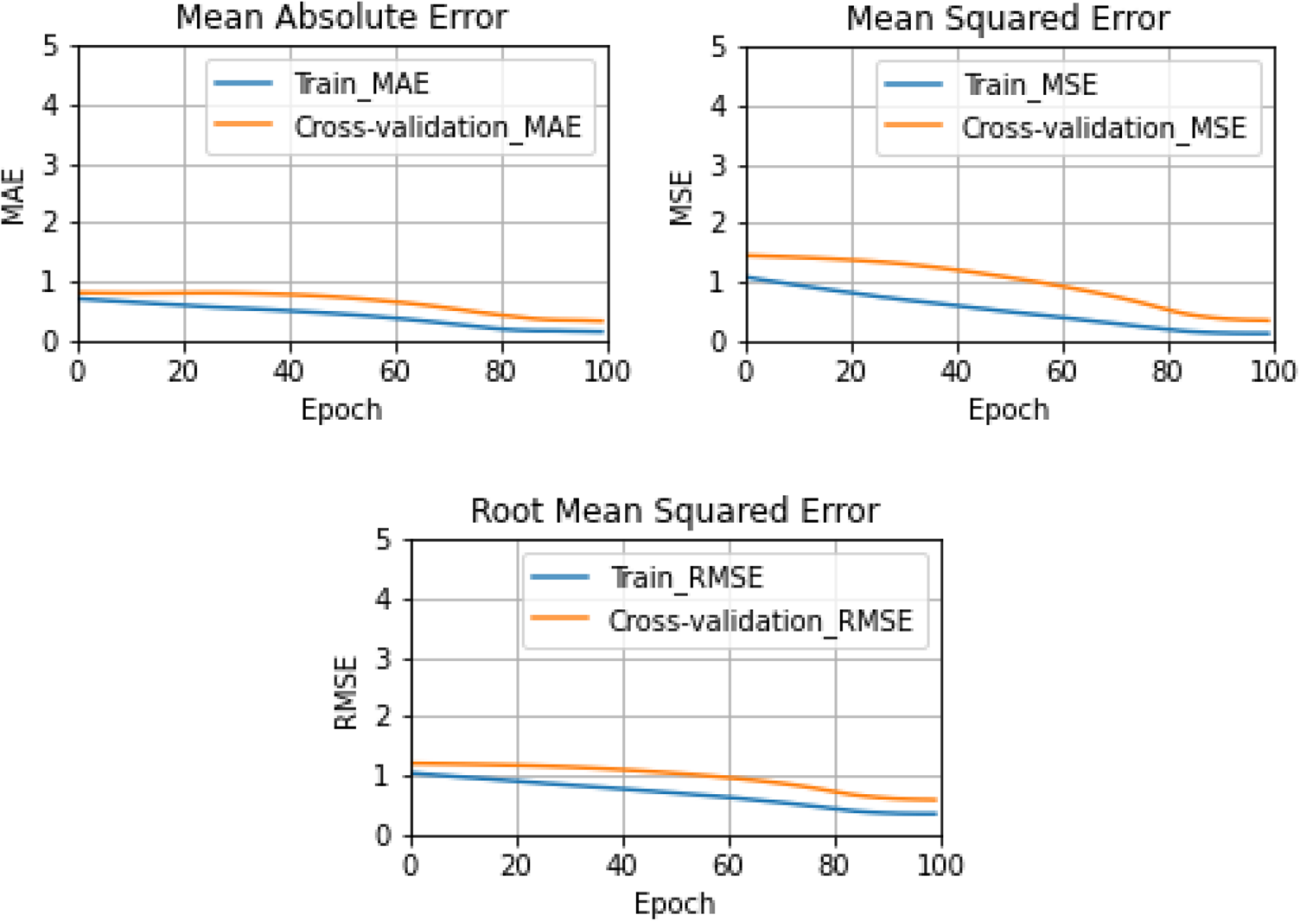
Performance evaluation on attention-assisted 1D CNN-BiLSTM model: MAE, MSE, and RMSE.

All the values of performance evaluation matrices are summarized in Table 6. It is clear from Table 6 that the values are almost equal to their preferable values. Based on the preferable values of all the performance matrices, it can be said that the proposed attention-assisted 1D CNN-Bi-LSTM model shows the superiority in prediction of induced electric field with a minor number of errors in the testing data. Moreover, Fig. 4 plots the training process of proposed model in terms of MAE, MSE, and RMSE against 100 iterations (epochs), in which the finest result is achieved after 80 epochs for the evaluation of regression performance.

**Table 6:** Performance evaluation matrices for electric field prediction task.

R^2^	MSE	MAE	RMSE
0.9992	0.0005	0.0188	0.0228

**Figure 5:**
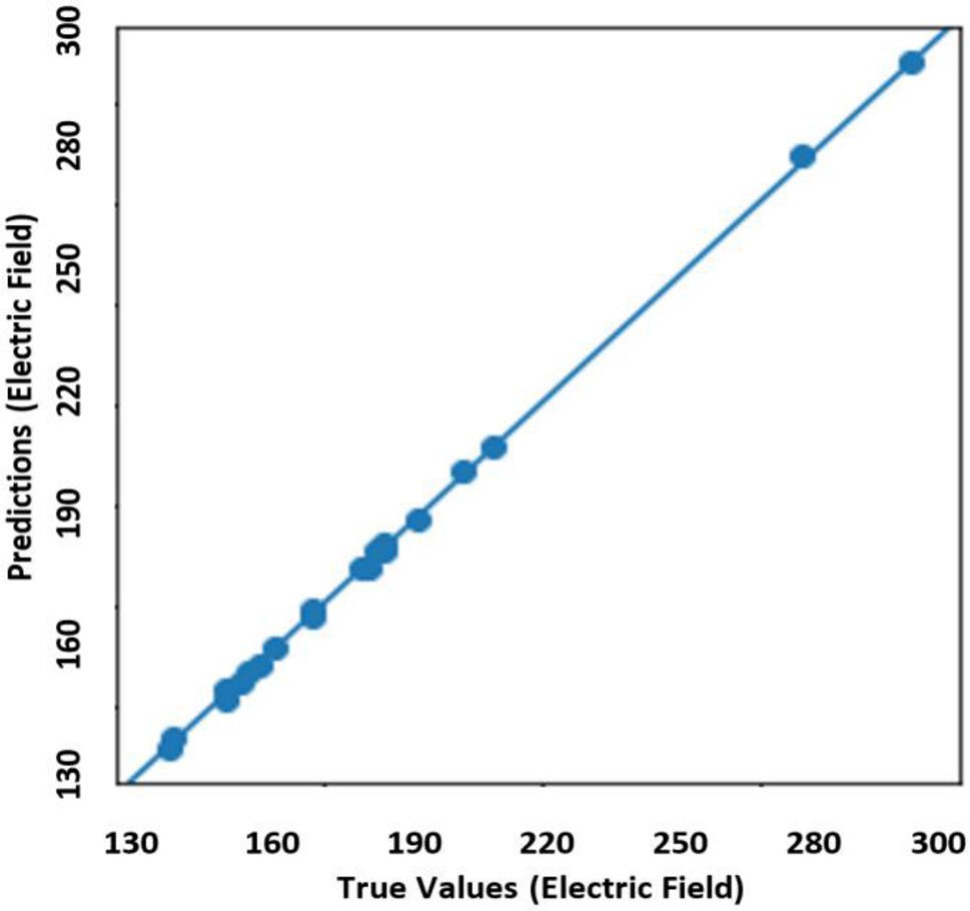
Scatter plot of predicted electric field values versus actual data on the test set.

**Figure 6:**
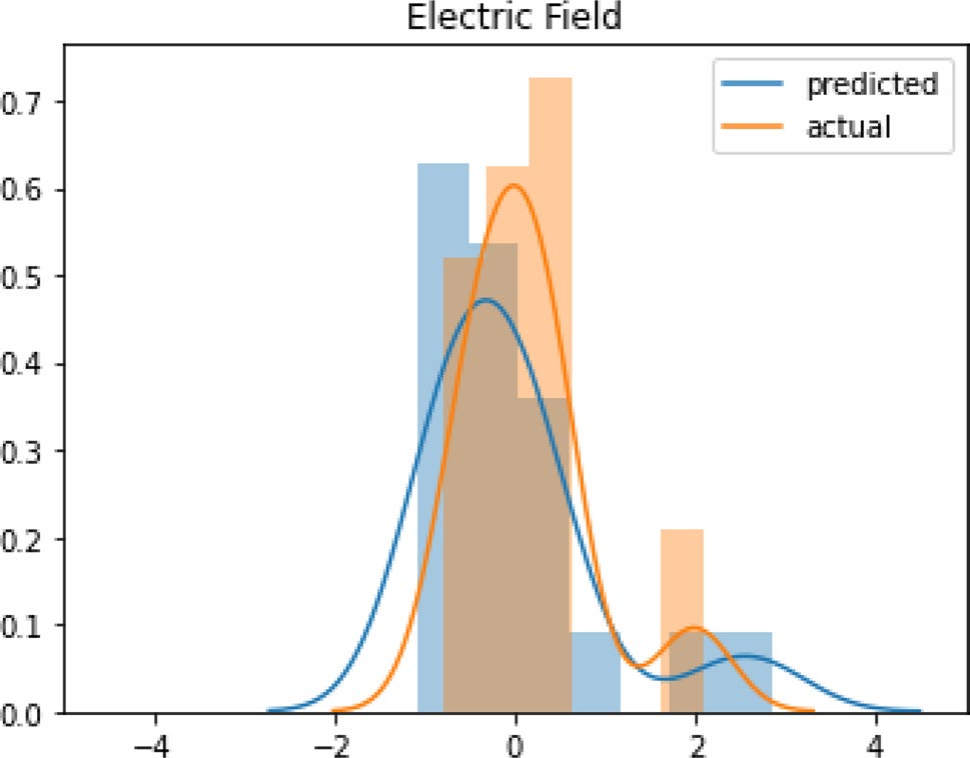
KDE probability density curve of the electric field: actual vs. predicted.

Figure 5 represents the scatter plot of predicted electric field values against simulated (ground truth) electric field values for the proposed attention-based model. From this graph, it can be observed that all the predicted electric field values are very close to the simulated electric field values that ensure good accuracy between the prediction from attention-based model and simulated data. For this reason, the proposed model is capable of enumerating induced electric field values in a correct manner ranging from 130 to 300 V/m. Moreover, the Kernel Density Estimation (KDE) is illustrated in Fig. 6 to estimate and compare the predicted electric field values with the actual electric field values on the test set in a probability density distribution. According to the performance of the prediction model, the KDE probability density curve of predicted electric field values agrees well with the actual electric field values. Thus, it is recognized that the proposed prediction model attains a superior performance on the test set.

## Discussion

**Figure 7:**
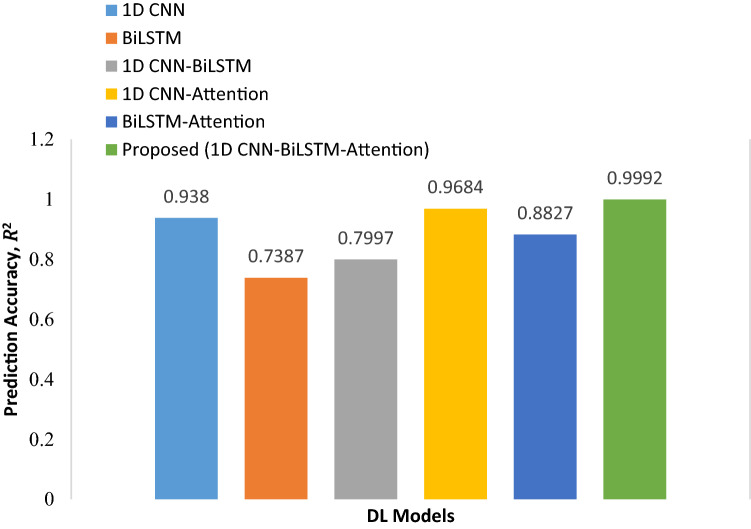
The prediction accuracy of different models on the validation dataset.

The prediction accuracy, ${R}^{2}$ of different possible regression models such as 1D CNN, BiLSTM, 1D CNN-BiLSTM, 1D CNN with attention, and BiLSTM with attention on validation dataset are illustrated in Fig. 7. Among these DL models, the proposed model provides the highest electric field prediction accuracy of approximately 99.92%. In addition, the model performance is evaluated by comparing the proposed attention-based hybrid model with the state-of-the-art models as presented in Table 7. The works reported in Refs.^15,16^ used a single TMS coil type with a single coil parameter of coil position to enumerate the electric field. The main drawback of using a single TMS coil is a lack of tradeoff between focality and depth of stimulation^10^. To ensure an efficient clinical treatment with minimum side effects, the finest tradeoff between focality and depth is needed. In another study^17^, the prediction of electric field from an assembly coil with different coil parameters is presented that meets the simulation tradeoff between depth and focality. However, the prediction model used in that work provides lower prediction accuracy in terms of R^2^ value which is approximately 0.766. Moreover, other evaluation matrices such as MSE, RMSE, and MAE that represent losses of the prediction model are quite larger. For this reason, this research work mainly concerns the improvement of model performance without compromising other required parameters. Compared to the existing works, the proposed attention-based hybrid deep CNN-BiLSTM model ensures optimum prediction accuracy of 0.9992 in terms of R^2^ value with the lowest computation time of 0.11 s. Though the database is created based on a spherical human head model rather than a realistic head model, the proposed 1D model prediction performance with a minor variation could ensure optimum therapeutic efficacy including effectiveness and safe stimulation during treatment pertaining to several neurological disorders.

**Table 7:** Comparison table of the proposed model with the existing related works.

Reference	^15^	^16^	^17^	This work
Prediction model	U-Net	Deep CNN	DNN	1D CNN-BiLSTM with Attention Mechanism
Data type	Unstructured: image	Unstructured: image	Structured: numerical value	Structured: numerical value
Number of datasets	261,072	800	100	100
Coil type	Single	Single	Assembly	Assembly
Coil parameter	Coil position	–	Coil position	Coil position
			Coil turns	Coil turns
			Coil thickness	Coil thickness
			Coil diameter	Coil diameter
			Coil angle	Coil angle
			Coil wings distance	Coil wings distance
Prediction task	Segmentation	Regression	Regression	Regression
Computational time	0.541 s	–	0.04 s	0.11 s
Performance evaluation matrix	CC = 0.93 PSNR = 29 dB MAE = 6 RMAD = 6%	R^2^ = 0.92 MAPE = 6.2%	R^2^ = 0.766 MSE = 0.184 MAE = 0.262 RMSE = 0.429	R^2^ = 0.9992 MSE = 0.0005 MAE = 0.0188 RMSE = 0.0228

## Conclusion

This work presents a regression model based on an attention-assisted hybrid 1D CNN-BiLSTM network for predicting electric fields induced by HVA TMS coil. Without compromising the computational cost of electric field enumeration, the model improves the prediction accuracy by employing attention layer in the CNN-BiLSTM model. The attention mechanism helps the model to predict electric field values from 130 to 300 V/m by impacting the most relevant parameters of the HVA coil. By feeding a lower-sized database of 100 samples, the model obtained reasonable prediction accuracy of R^2^ = 0.9992 that ensures the capability of the model to enumerate electric field from HVA coil with varied coil designing parameters. Without requiring any three-dimensional mathematical model of human head phantom and TMS coil, the proposed method efficiently estimated electric field from a new unknown dataset in a very short time of 0.11 s. Therefore, to meet the requirement of the neurological disorder patients, the proposed attention-based model can aid the TMS manufacturer to design an optimum coil based on the predicted electric fields. In the future, a database with larger samples can be developed by considering several new assembly coils rather than single assembly coils to improve model generalization capability. Moreover, database can be developed based on the numeric data from the realistic anatomical head model to increase the practical feasibility of the work.

## Data Availability

The datasets generated during and/or analysed during the current study are available from the corresponding author on reasonable request.
